# Auditory Categorization of Man-Made Sounds Versus Natural Sounds by Means of MEG Functional Brain Connectivity

**DOI:** 10.3389/fnins.2019.01052

**Published:** 2019-10-04

**Authors:** Vasiliki Salvari, Evangelos Paraskevopoulos, Nikolas Chalas, Kilian Müller, Andreas Wollbrink, Christian Dobel, Daniela Korth, Christo Pantev

**Affiliations:** ^1^Institute for Biomagnetism and Biosignalanalysis, University of Münster, Münster, Germany; ^2^School of Medicine, Faculty of Health Sciences, Aristotle University of Thessaloniki, Thessaloniki, Greece; ^3^Department of Otorhinolaryngology, Friedrich-Schiller University of Jena, Jena, Germany

**Keywords:** auditory perception, functional connectivity, magnetoencephalography, sound discrimination, man-made sound, natural sound

## Abstract

Previous neuroimaging studies have shown that sounds can be discriminated due to living-related or man-made-related characteristics and involve different brain regions. However, these studies have mainly provided source space analyses, which offer simple maps of activated brain regions but do not explain how regions of a distributed system are functionally organized under a specific task. In the present study, we aimed to further examine the functional connectivity of the auditory processing pathway across different categories of non-speech sounds in healthy adults, by means of MEG. Our analyses demonstrated significant activation and interconnection differences between living and man-made object sounds, in the prefrontal areas, anterior-superior temporal gyrus (aSTG), posterior cingulate cortex (PCC), and supramarginal gyrus (SMG), occurring within 80–120 ms post-stimulus interval. Current findings replicated previous ones, in that other regions beyond the auditory cortex are involved during auditory processing. According to the functional connectivity analysis, differential brain networks across the categories exist, which proposes that sound category discrimination processing relies on distinct cortical networks, a notion that has been strongly argued in the literature also in relation to the visual system.

## Introduction

Current knowledge on neuronal networks underlying auditory perception remains fragmentary, despite the fact that audition has been extensively studied (Zatorre et al., 2002). The basic network properties that have been suggested for the auditory modality resemble the structure of the visual one, dividing the information processing pathways in dorsal and ventral networks, corresponding to the processing of information to “where” and “what,” respectively (Romanski et al., 2000; Kubovy and Van Valkenburg, 2001; Arnott et al., 2004; Huddleston et al., 2008; Asplund et al., 2010). Following the “what” pathway, the physical characteristics of the sound stimulus are initially encoded in the primary and secondary auditory cortex, along with their associative areas, prior to their integration into a more abstract representation (Griffiths and Warren, 2004; Bregman, 2017). Within this pathway, the processing of auditory information seems to be performed in sound category specific channels (Caramazza and Mahon, 2003). In this line, suggestions have been raised to propose functional specificity for processing different types of sounds (Belin et al., 2000; Zatorre and Belin, 2001; Patterson et al., 2002; Lewis et al., 2004; Zatorre et al., 2004; Hunter et al., 2010).

Attempts to dissociate the processing of different sound categories at the cortical level have been made in brain-lesion case studies (Clarke et al., 2000; Taniwaki et al., 2000; Mendez, 2001; Steinke et al., 2001). Cases like auditory agnosia, represent the impaired ability to recognize sounds, when peripheral hearing is intact. However, this impairment does not necessarily apply to all sound categories; it may rather be category-specific depending on the brain damage. For instance, a patient with focal damage in the right fronto-parietal area was able to identify environmental sounds and name musical instruments, but could not recognize music (Steinke et al., 2001). On the other hand, left temporal lesion or left fronto-temporal ischemia have caused agnosia restricted to environmental sounds (Clarke et al., 2000). It should be emphasized though, that single-case studies of brain-lesioned patients are very heterogeneous and therefore they cannot provide a detailed model of cortical sound processing.

Several functional neuroimaging studies with healthy participants indicate bilateral auditory cortex activation for speech sounds (Belin et al., 2000; Zatorre and Belin, 2001) and right-lateralized activation for non-speech sounds during sound discrimination tasks (for review see Tervaniemi and Hugdahl, 2003). Other regions of the brain, such as the inferior frontal cortex have been also reported to be involved, indicating a network that involves further cognitive functions, beyond the auditory ones (for review see Price, 2012). So far, the majority of current studies are mainly focused in the differential processing of speech versus non-speech sound categories, though we still have poor knowledge about the differential processing within non-speech sound category. The existing studies have shown that sound category discrimination depends more likely on its associated manipulative characteristics (Lewis et al., 2004, 2005; Murray et al., 2006; De Lucia et al., 2009, 2012). For instance, in the context of playing a guitar, we listen to the sound while we perceive motor and visual actions of the guitar playing. Thus, at a higher cognitive level information from all sensory modalities that receive input from a stimulus are integrated in order to construct the percept of the sound. Similar model (Heekeren et al., 2008) has been proposed in the past for the visual and somatosensory system, indicating a multisensory integration already in low level early stages of cognitive processing.

Evidence on the functional organization of auditory perception shows that sounds can be categorized into “living” and “man-made” stimuli (Lewis et al., 2005), suggesting differential brain activation. In particular, a man-made object in comparison to a sound of an animal, might require a top-down mechanism which integrates semantic and multisensory features associated more with action. Similarly, Murray et al. (2006) demonstrated by means of EEG, that “man-made” sounds display stronger brain activation in the auditory “what” pathway compared to the “living” objects, and that regions of the right hemisphere and premotor cortices were mainly involved. Other differentiations have been also reported within the category of man-made objects. The main idea is that daily used object sounds, such as the phone ringing, might trigger more response of action than a typical tone of a musical instrument (in non-musicians) and, thus stronger brain activation will be elicited (De Lucia et al., 2009). Interestingly, EEG studies focusing on the temporal dynamics have shown that the category discrimination process occurs around the N1 component, already 70 ms after the stimulus onset (Murray et al., 2006; De Lucia et al., 2012).

Nevertheless, the way distinct networks operate across different categories of sounds is still poorly understood. Although, the aforementioned studies have given some insights of when and where this differentiation appears, the source space analyses offer simple maps of activated brain regions, rather than indicating how these regions of a distributed system are functionally connected to execute a specific task. Up to date, the investigation of complex networks has been developed methodologically, given the opportunity to study cortical reorganization underpinning associated cognitive processes (Rogers et al., 2007; Bullmore and Sporns, 2009). Therefore, in the current study we aimed to further investigate the functional connectivity of the auditory processing pathway across different non-speech sound categories. The cortical responses of three different categories of sounds were compared. Namely, the *Musical* and the *Artificial* category (sounds of daily used/heard objects), representing the man-made-objects sound categories and the *Natural* category (mainly animal vocalizations). According to our knowledge, this is the first study to investigate functional connectivity across different non-speech sound categories by means of magnetoencephalography (MEG), which has high spatial resolution and excellent temporal resolution. Taking into consideration the existing literature about living versus man-made-related sounds, we expected that the *Musical* and the *Artificial* sounds would demonstrate stronger cortical responses, that would involve motor related regions and significant interconnections among these regions in comparison to the *Natural* sounds. Further, it should be noted that although the *Musical* and the *Artificial* sounds belong to the man-made category, evidence for differential activation between these groups has been previously reported (De Lucia et al., 2009) as a function of daily use. The N1 auditory evoked field was *a priori* set as the time interval of interest based on previous electrophysiological findings that report early responses in the sound category discrimination processing (Murray et al., 2006; De Lucia et al., 2009, 2012).

## Materials and Methods

### Subjects

The current study was conducted with a sample of 20 young adults (mean age = 27.19, SD = 5.59, 8 males). They were recruited from the pool of subjects of our institute among those who had normal hearing, according to a clinical audiometric evaluation. All subjects were right handed, according to the Edinburgh Handedness Inventory (Oldfield, 1971). The participants were informed about the aim of the study and the ones willing to participate were provided with a consent form that ensured the confidentiality of their identity. The study was performed according to the Declaration of Helsinki and approved by the ethics committee of the Medical faculty of the University of Münster.

### Stimuli

The stimuli consisted of three different categories of sounds: *Natural*, *Musical*, and *Artificial*. The *Natural* and *Artificial* sounds were recordings obtained from online sound databases (Free Sounds Effects^1^; SoundBible^2^; ZapSplat^3^). The *Musical* sounds were obtained from “McGill University Master samples” sound bank that have been created for perceptual research related to the psychology of music. The Audacity software^4^ was used to resample all sounds at 44,100 Hz and to implement onset/offset linear slopes of 20 ms. Then, the mono sounds were converted into stereo sounds. By means of the WavePad Sound Editor^5^ they were normalized by −10 dB RMS based on the Average Loudness normalization method.

The stimulus paradigm was performed via Presentation software (Version 18.0, Neurobehavioral systems, Inc., Berkeley, CA, United States)^6^. It consisted of two blocks with a short break in between. Each block included the presentation of the three different categories of sounds that were pseudo-randomly presented across blocks and across subjects, whereas the sounds of each category were presented always in the same order: A = *Artificial*, M = *Musical* and N = *Natural*; Block1: A1-A2-…-An-M1-M2-…-Mn-N1-N2-…-Nn; Block2: M1-M2-…-Mn-N1-N2-…-Nn-A1-A2-…-An. Each block contained 144 stimuli, 48 for each category, that makes a total of 288 stimuli for the whole experiment; 48 (sounds per category) × 3 (categories) × 2 (blocks). Each block contained 144 stimuli, 48 for each category, that makes a total of 288 stimuli for the whole experiment; 48 (sounds per category) × 3 (categories) × 2 (blocks). Each stimulus lasted for 1 s with a randomized Inter-Stimulus Interval (ISI) between 0.7 and 1.3 s, in order to avoid expectancy and rhythmicity. The *Natural* sounds contained sounds of living objects. The *Musical* sounds contained notes of different musical instruments, whereas the *Artificial* sounds were daily object-like sounds. Examples of the sound files used in the study can be found in the Supplementary Material.

### MEG Recordings

Participants were examined in a magnetically shielded and acoustically quiet room by means of 275 channel whole-head system (OMEGA 275, CTF, VSM Medtech Ltd., Vancouver, BC, Canada). Data were continuously recorded with a sampling frequency of 600 Hz resulting in an off-line cut-off frequency of 150 Hz. Participants were seated in an upright position and their head was stabilized with cotton pads inside the MEG helmet. A silent movie was presented on a projector screen mounted on the MEG system gantry, placed according to participants’ best view angle, in order to keep them staying vigilant during the experiment; as been applied in previous auditory experiments (Pantev et al., 2004; Okamoto et al., 2008; Paraskevopoulos et al., 2018). After passing electro-static transducers the auditory stimuli were delivered via silicon tubes of 60 cm length and an inner diameter of 5 mm ending with a silicon earpiece fitted individually to each subject’s ear. Prior to the stimulation, an audiological hearing threshold determination test with 5 dB accuracy on 1 kH frequency, was conducted. Stimulus sound pressure levels were set to 60 dB SL above the individual hearing threshold. The whole experiment lasted for approximately 30 min.

### MRI Protocol

A T1-weighted MR image was performed for all participants, in a 3 Tesla scanner (Gyroscan Intera T30, Philips), in order to obtain the individuals’ Finite Element Model (FEM) of the head. The files gave images of 400 one layer-slices with 0.5 mm thickness in the sagittal plane (TR = 7.33.64 ms, TE = 3.31 ms). The matrix size of each slice was 512 × 512 with voxel size of 0.5 × 0.58 × 0.58 mm^3^. To ensure the reliability of investigation on brain structure within and across subjects, we used SPM12 (Statistical Parametric mapping)^7^ to regulate for intensity inhomogeneity (Ganzetti et al., 2016) and therefore, the images were resliced to isotropic voxels of 2 × 2 × 2 mm.

### MEG Data Analysis

The analysis of the MEG data was run according to a previously developed analysis applied for functional connectivity networks under different auditory paradigms (Paraskevopoulos et al., 2015, 2018). The Brain Electrical Source Analysis software (BESA MRI, version 2.0, Megis Software, Heidelberg, Germany) was used to compute the individual’s head model by segmenting four different head tissues (scalp, skull, CSF, and brain), based on the FEM. The four-layer FEM model gives more precise results as compared to other models, since it includes the CSF (Ramon et al., 2004; Wendel et al., 2008), which is a highly conductive layer and important for MEG source reconstruction (Wolters et al., 2006). The MEG sensors were co-registered and adjusted to the individuals’ structural MRI via the nasion, and the left and the right entries of the ear-canals as landmarks. By means of 3D spline interpolation the MRIs were transformed to ACPC (anterior-posterior cingulate) and to Talairach space. A predefined option for conductivity values (c.f. Wolters et al., 2006) was set for the skin compartment to 0.33 S/m, for the skull to 0.0042 S/m, for the CSF to 0.79 S/m and for the brain tissue to 0.33 S/m.

For the pre-processing of the MEG data, the BESA research software (version 6.0, Megis Software, Heidelberg, Germany) was used. For artifact rejection, an automated electrocardiogram (ECG) and eye blinks artifact detection and correction provided by BESA (Ille et al., 2002) was applied. Data were filtered off-line with a 50 Hz notch filter, zero-phase low-pass filter of 45 Hz and zero-phase high-pass filter of 0.5 Hz. The data were divided into epochs of 1000 ms post- and 500 ms pre-stimulus onset. A baseline correction based on a 100 ms pre-stimulus interval was applied. During the averaging of the stimulus related epochs, trials having amplitudes larger than 3 pT and data exceeding the 15% of rejected trials, were excluded from the analysis. The two measurement blocks were then averaged for each participant in order to improve the signal-to-noise ratio.

For the current density reconstruction, we used a time window around the N1 major component of the slow auditory evoked field (Pantev et al., 1993; c.f. Figure 2), which according to the global field power of the grand average data was between 80 ms and 120 ms after stimulus onset, including the rising slope of the N1. Low Resolution Electromagnetic Tomography (LORETA) provided by BESA was applied for the source reconstruction, for each subject and each category of sounds as it provides smooth distribution of sources as inverse solution (Pascual-Marqui et al., 1994). It is based on the weighted minimum norm method (Grech et al., 2008) and it does not rely on an *a priori* determination of activated sources.

### Statistical Analysis

For the statistical analysis of the LORETA reconstruction, we used the SPM12 running on Matlab software (R2016b version; MathWorks Inc., Natick, MA, United States). An explicit mask was set, to include results only for the gray matter, thus decreasing the search volume. One-way ANOVA analysis was run with the three different categories of sounds (*Natural*, *Artificial*, and *Musical*) as within-subjects factor. F- and t-contrast matrix-tables were then designed (based on the general linear model) to test for statistical differences across the three categories and between-categories, respectively. For multiple comparisons control, the Family Wise Error (FWE) was implemented.

### Connectivity Analysis

In order to examine the cortical network across the significant sources derived from the SPM12 analysis, we further implemented a connectivity analysis. Having defined the activated regions in source space via the above described analysis, we employed an equivalent current dipole model by setting one dipole to the peak of each significant cluster derived by the F-contrast. This resulted into five equivalent current dipoles in total. Due to the fact that SPM expresses coordinates based on standardized brains by the Montreal Neurological Institute (MNI coordinates), the coordinates were transferred to Talairach space to fit the brain coordinates of BESA software, where the dipole model was run. For the conversion the “NMI2TAL” applet of the Yale BioImage Suite Package was used (sprout022.sprout.yale.edu), which is based on the Lacadie et al.’s (2008) mapping coordinates. The orientation of the dipoles was fitted based on the individuals FEM volume conductor, whereas the coordinates were fixed across all subjects and conditions, as defined above. The results contained five source waveforms that corresponded to each dipole including the 80–120 ms interval.

**FIGURE 1:**
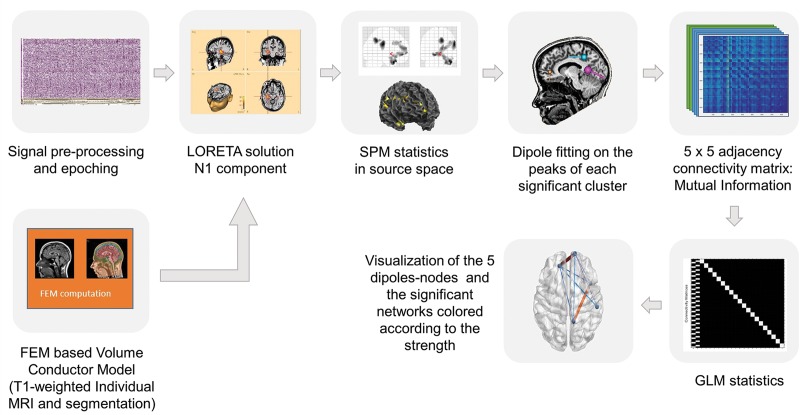
Pipeline of the analysis tools. First step: The individual MRI segmentation based on FEM volume conductor model was computed with the BESA MRI software. Pre-processing of the time series and averaging of the evoked fields were performed in BESA research software and for the source reconstruction the LORETA solution has been applied. Second step: The SPM statistics toolbox in Matlab was used for statistical analysis of the data. Third step: For the connectivity analysis equivalent current dipoles were set to the significant regions revealed by the previous analysis. Fourth step: HERMES toolbox in Matlab was used to construct a 5 × 5 adjacency matrix for each subject and each condition based on the Mutual Information algorithm. Fifth step: The statistical analysis of the functional connectivity was performed in the NBS toolbox and the data were transferred to the BrainNet toolbox to visualize the statically significant connectivity networks.

The HERMES toolbox (Niso et al., 2013) of Matlab was used to construct a 5 × 5 adjacency matrix for each subject and each condition based on the Mutual Information (MI) algorithm, which measures the mutual dependence between variables and it detects correlations of random variables with non-linear dependence measure (Zeng, 2015). The results were then transferred to the Network Based Statistic Toolbox (NBS; Zalesky et al., 2010) to examine statistically significant connections. One way within-subjects ANOVA was run with the three conditions as the within-subjects factor. The NBS method was set for multiple correction at the significant level of *p* > 0.05 (see Figure 1 for analysis pipeline tools). This resulted in a functional connectivity graph with nodes and edges representing the significant activated regions and their significant interconnection, respectively.

## Results

### Source Space

**FIGURE 2:**
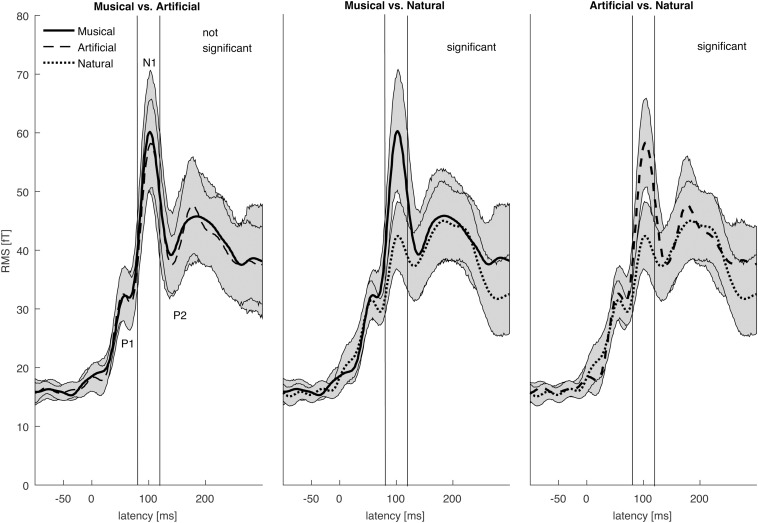
The root mean square of the grand averages of the auditory evoked field (AEF) for the *Musical*, *Artificial*, and *Natural* sounds. The graphs illustrate the brain activation within the time domain (–100 pre-stimulus onset to 300 ms post-stimulus) with 0 ms representing the stimulus onset. The AEF components P1, N1, and P2 as well the time interval 80–120 ms are labeled. The means of the different types of sounds are marked with different types of lines. Their corresponding 95% confidence intervals are given by shaded areas. The time interval of interest is around the N1 component of the auditory evoked field (80–120 ms).

**FIGURE 3:**
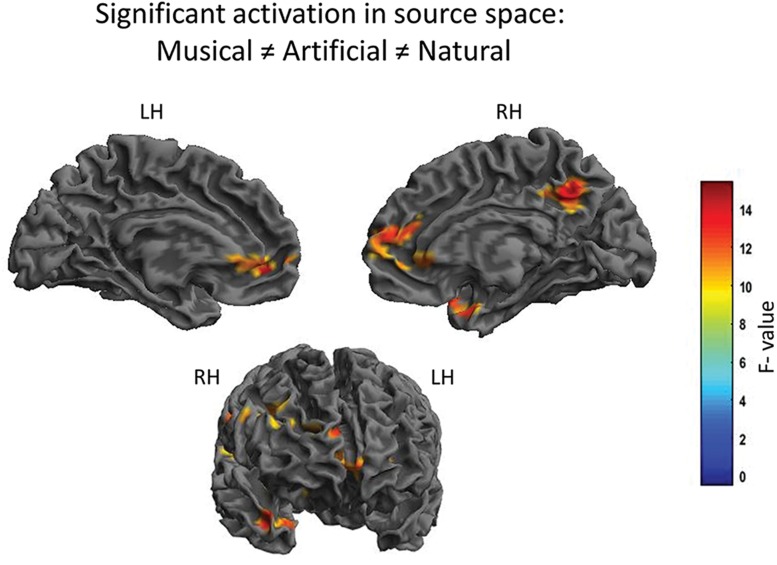
Significant differential cortical activation in source space based on the F-contrast. The figures depict the statistical parametric mapping as derived from the one-way ANOVA analysis of the SPM statistics for the *Musical* ≠ *Artificial* ≠ *Natural* interaction. At the top, on the **left** side the midsagittal view of the **left** hemisphere; **top-right** side the midsagittal view of the **right** hemisphere; at the bottom the frontal view are presented. The **left** and **right** prefrontal cortex and the anterior STG, as well as, the supramarginal gyrus and the PCC (overlapping in the figure) are activated. The color scale represents the *F*-values at *p* < 0.001 level of significance (FWE corrected).

The N1 auditory evoked field was *a priori* set as the time window of interest. The root-mean square time series of the grand average across subjects was computed in sensor space to depict the time window in ms around the *a priori* set N1 auditory field maximum. The Figure 2 illustrates the mean of the root-mean square values of each sound category as well as the maximum and minimum values of their confidence intervals. The 80–120 ms interval was determined for the following source reconstructions where we performed F- and t- contrast statistics by means of the SPM12 software. Figure 3 and Table 1 illustrate the significant clusters obtained by the *Musical* ≠ *Artificial* ≠ *Natural* contrast. The biggest in size cluster involved parts of the right and left frontal cortex, as well as, parts of the temporal lobe. The peak of the current cluster was located in the anterior part of the right temporal cortex, in the most dorsal area of the superior temporal gyrus (STG) (*x* = 43, *y* = 14, *z* = −29; *F*(1, 20) = 13.1, cluster size = 3128, *p* < 0.001 FWE corrected at cluster level). A second cluster was located in the right inferior parietal lobe, with the peak in the right supramarginal gyrus (SMG) (coordinates: *x* = 56, *y* = −27, *z* = 27; *F*(1, 20) = 12.80, cluster size = 513, *p* < 0.001 FWE corrected at cluster level). The third cluster was located in the posterior cingulate cortex (PCC; overlapping with cluster two in the figure), which involved the posterior cingulate gyrus and medial part of the parietal lobe (coordinates: *x* = 10, *y* = −52, *z* = 39, *F*(1, 20) = 12.42, cluster size = 650, *p* < 0.001 FWE corrected at cluster level).

**TABLE 1:** Significant clusters based on the F-contrast.

**Location of cluster**	**MNI coordinates**			**Cluster size in voxel**	***F*(1, 20)**	**P FWE-corrected**
	***X***	***Y***	***Z***			
Right aSTC	43	14	−29	3128	13.01	<0.001
Right SMG	56	−27	27	513	3.86	<0.001
PCC	10	−52	39	650	3.81	<0.001

*The locations of clusters with corresponding MNI coordinates of significant different cortical regions as derived by the source space analysis for the *Musical* ≠ *Artificial* ≠ *Natural* contrast are given. Cluster size in voxel, *F*- and *p*-values are also depicted, FEW corrected at *p* < 0.001 level of significance.*

**FIGURE 4:**
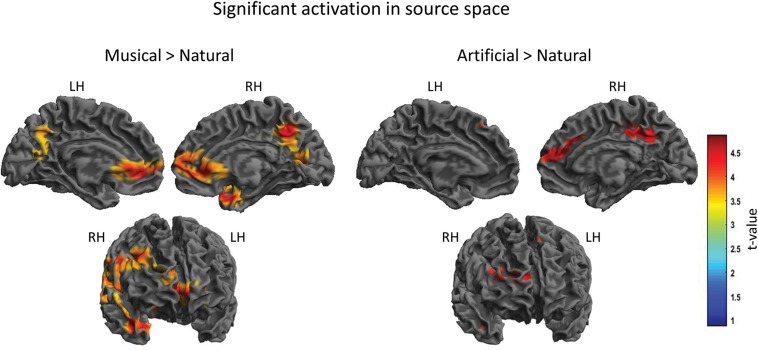
Significant cortical activation in source space of the between-subject comparison. The figures depict the statistical parametric mapping as derived from the *t*-tests analysis of the SPM statistics for the *Musical* > *Natural* and *Artificial* > *Natural* comparisons. At the top, on the **left** side the midsagittal view of the **left** hemisphere; **top-right** side the midsagittal view of the **right** hemisphere; at the bottom the frontal view are presented. The **left** and **right** prefrontal cortex and the anterior STG, as well as, parts of the **left** and **right** PCC and the supramarginal gyrus (overlapping in the figure) are activated as illustrated in the panel on the **left** (*Musical* > *Natural*). The panel on the **right** (*Artificial* > *Natural*) displays the activated parts of the **right** prefrontal cortex, the supramarginal gyrus and the PCC. The color scale represents the *t*-values at *p* < 0.001 level of significance (FWE corrected).

For the between groups comparisons, the results revealed significant differences for both *Musical* > *Natural* and *Artificial* > *Natural* comparisons as demonstrated in Figure 4 and Table 2, depicting the coordinates and the source mapping results for both comparisons. In more detail, two significant clusters were obtained for *Musical* > *Natural* sounds. The biggest cluster involved regions of left and right frontal cortex, as well as, temporal cortex and SMG. The peak of the current cluster was located at the SMG (*x* = 56, *y* = −27, *z* = 27, *t*(20) = 5.05, cluster size: 109960, *p* > 0.001 FWE corrected at cluster level). The second cluster was located in the PCC (coordinates: *x* = 8, *y* = −54, *z* = 40, *t*(20) = 4.61, cluster size = 3142, *p* < 0.001 FWE corrected at cluster level). For the *Artificial* > *Natural* contrast we found two clusters revealing significant differential activation; one significant peak was located at the PCC (coordinates: *x* = 10, *y* = −37, *z* = 39, *t*(20) = 4.09, cluster size = 675, *p* = 0.001 FWE corrected at cluster level) and the other one at the medial prefrontal cortex mPFC (coordinates: *x* = 13, *y* = 39, *z* = 18, *t*(20) = 4.08, cluster size = 1007, *p* < 0.001 FWE corrected at cluster level).

**TABLE 2:** Significant clusters of the between-subject comparisons.

**t-Contrast**	**Location of cluster**	**MNI coordinates**			**Cluster size in voxel**	***t*(20)**	**P FWE-corrected**
		***x***	***y***	***z***			
*Musical*> *Natural*	Right SMG	56	−27	27	109960	5.05	<0.001
	PCC	8	−54	40	3142	4.61	<0.001
*Artificial*> *Natural*	Right	13	39	18	1007	4.08	<0.001
	mPFC						
	PCC	10	−37	39	675	4.09	0.001

*The location of clusters with the corresponding MNI coordinates of the significant different cortical regions as derived by the source space analysis for the *Musical-versus-Natural* and *Artificial-versus-Natural t*-tests are shown. Cluster size, *t*- and *p*-values are also depicted, FEW corrected at *p* < 0.001 level of significance.*

### Connectivity Results

For the connectivity analysis, five equivalent current dipoles were set to the peaks of the significantly differential activated areas as derived from the F- contrast. These are the PCC, the aSTG, the SMG and the bilateral prefrontal cortex. Three connectivity analyses were conducted based on the significant interaction found in the source space analysis, *p* < 0.05, NBS corrected (Network-Based Statistics; Zalesky et al., 2010).

**FIGURE 5:**
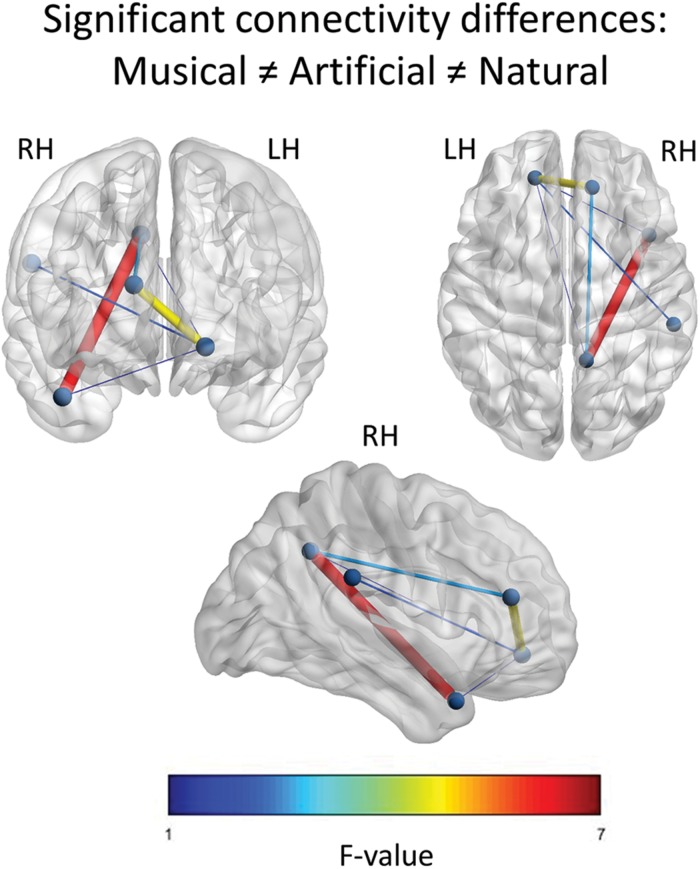
Significant F-contrast functional connectivity network. The statistically significant connections across the nodes, for the *Musical* ≠ *Artificial* ≠ *Natural* contrast are illustrated. The nodes represent the significant regions revealed by the source space analysis, that were set as equivalent current dipoles. The edges are weighted and colored, according to their connectivity strength, as indicated by the *F*-value of the color scale. The networks are significant at *p* < 0.05, NBS corrected. The **upper-left** figure depicts the frontal coronal view, the **upper-right** one displays the axial plane viewed from the top and the figure at the bottom illustrates the right sagittal plane.

For the *Musical* ≠ *Artificial* ≠ *Natural* contrast, the results revealed connections of all the nodes having six edges in total. As Figure 5 demonstrates, edges between PCC and right anterior superior temporal gyrus (aSTG), as well as, between left ventral mPFC and right medial dorsal prefrontal cortex had the strongest activation (as indicated by the *F*-value). The left mPFC yielded significant differential connections with all the nodes located in the right hemisphere and it was the only one connecting with the SMG.

**FIGURE 6:**
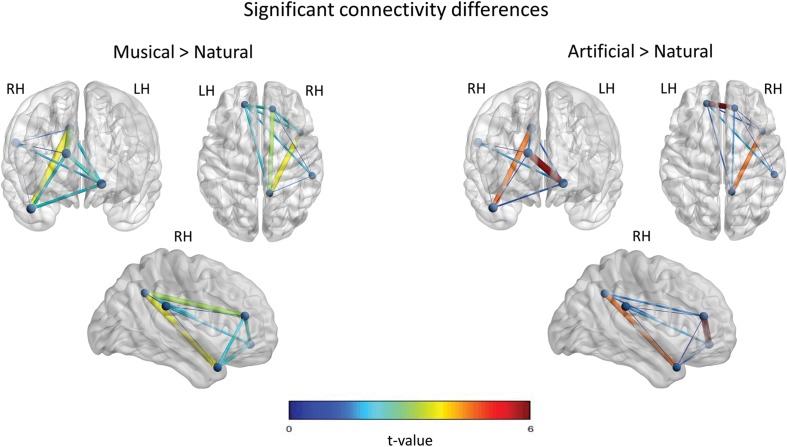
Significant functional connectivity network of the between-subject comparisons. The statistically significant connections across the nodes, for the *Musical* > *Natural* and *Artificial* > *Natural* comparisons are illustrated, respectively. The nodes represent the significant regions revealed by the source space analysis, that were set as equivalent current dipoles. The edges are weighted and colored according to the connectivity strength, as indicated by the *t*-value of the color scale. The networks are significant at *p* < 0.05, NBS corrected. The **upper-left** figure in both, left and right panels depicts the frontal coronal view, the **upper-right** figure the axial plane viewed from the top, and the figure at the bottom illustrate the right sagittal plane.

In the *Music-versus-Natural* comparison a significant differential network of 10 edges was yielded with all the nodes in the network being interconnected (c.f. Figure 6). According to the *t*-value, increased functional connectivity was demonstrated between the right mPFC and the PCC, as well as, between the PCC and the aSTG. With smaller *t*-value, the left mPFC yielded significant interconnections with the right PFC, the aSTG, the SMG and the PCC. Weaker interconnections were obtained between the SMG and the aSTG, the PCC, as well as the left mPFC.

With regard to *Artificial-versus-Natural* comparisons (c.f. Figure 6), the results showed significant differential interconnections among all the nodes having nine edges in total. In detail, the edge-connection of the prefrontal inter-hemisphere and the connection between the aSTG and PCC nodes, were the most pronounced according to the *t*-value. Slighter in strength edges were revealed for the rest of interconnections, but no significant interconnection was found between PCC and SMG.

## Discussion

The current study examined neural responses in the processing of different categories of sounds, by means of MEG. Further, functional connectivity analysis based on the MI algorithm was used to depict the differential connectome of the regions involved in the discrimination of *Natural*, *Musical*, and *Artificial* sound-category. The obtained results demonstrated statistically significant differences across the different sound conditions in the superior temporal cortex, the posterior cingulate, the inferior parietal and the bilateral prefrontal cortex, between 80 and 120 ms after stimulus onset. Direct comparisons across the different categories, showed that both *Musical* and *Artificial* sounds demonstrated statistically significant differences and more enhanced brain activation in source space and connectivity analysis, when compared to *Natural* sounds category.

Our finding that regions of parietal cortex showed significant category modulation of activation, as derived from the contrast analysis, it is consistent with several neuroimaging studies investigating the organization of cortical auditory perception (Lewis et al., 2005; Murray et al., 2006; Staeren et al., 2009). In our study, we found two regions in the parietal lobe to be involved in the differential processing of the assessed sound categories, namely, the PCC and the right inferior parietal cortex (IPC). The peak of the IPC cluster was located in the SMG, which is part of the somatosensory cortex (Buccino et al., 2001). In fact, it is suggested to be involved in action representation and specifically in the mental representation of movement (Chong et al., 2008; Tunik et al., 2008). Within the framework of this interpretation, any event or stimuli containing actions would engage motor related regions even in the absence of tactile stimulation (Nilsson et al., 2000). Additional evidence associates the SMG with the mirror neuron system, a system involved in imitating and identifying the actions of other persons (Carlson, 2012). On this basis, the right SMG has been suggested to be especially involved in motor planning, even when the actions are just observed and not necessarily executed (Chong et al., 2008). It seems that in the mental representation, information of how a tool is manipulated, visualized or how the sound is produced, are retrieved and integrated.

Apart from the parietal cortex, our connectivity results further stressed the involvement of the prefrontal cortex and the STG in the processing of different sound categories. Significant functional connectivity between prefrontal cortex and parietal cortex has been demonstrated in the past, suggesting that this network is part of the working memory network, linking perception and higher order cognition. This is rather multi-modal and regards both auditory and visual object processing (Husain et al., 2004; Husain and Horwitz, 2006). With respect to the STG, most of neuroimaging studies have shown the significant involvement of this region in the auditory processing. However, according to the meta-analysis of Arnott et al. (2004; including 38 studies of different neuroimaging techniques) it seems that the anterior area of the STG, as demonstrated here, is associated to greater extend with the discrimination of sounds. In line with our connectivity results, the STG region has been found to interact significantly with the prefrontal cortex during category discrimination task (Husain and Horwitz, 2006). The fact that our connectivity pattern was lateralized on the right hemisphere is consistent with the suggestion that this hemisphere is more strongly activated, when non-speech or non-living stimuli are represented (see Zatorre et al., 2002; Tervaniemi and Hugdahl, 2003). This derives not only from brain lesion studies, but also from neuroimaging studies with healthy participants (Belin and Zatorre, 2003; Murray et al., 2006).

Another possible interpretation of our results is that the processing of Musical and Artificial sounds involves more the default mode network (DMN). The PCC area, as derived by our results, has been strongly suggested to have a central role in the DMN (for review see Leech and Sharp, 2013) with the inferior parietal lobe, prefrontal cortex and temporal cortex (however, more medial structures) as the main nodes for intrinsic connections (Raichle et al., 2001). Activation of DMN has been mainly observed under task-free conditions when attention to external stimuli is not required (for review see Buckner et al., 2008). However, increased activity during cognitive processing which requires internal attention is also found, such as memory retrieval and planning (Spreng, 2012). From this view, the processing of man-made objects sounds might require a more top-down processing of information than the living-object sounds do. Moreover, the PCC has been highly associated with emotional processing and arousal state with higher responses and large-scale functional connectivity under arousal state and decreased on sleeping state (Leech and Sharp, 2013). As such, stimulation with Musical and Artificial sounds might evoke high-arousal emotions in relation to natural sounds. Nevertheless, the lack of a relevant behavioral measurements does not allow for further interpretation on this assumption and it is beyond the scope of this paper. In the future, a behavioral test assessing the levels of emotional arousal to different sounds would give us some insights on whether this factor affects significantly the differential brain activation.

Our main hypothesis was that the man-made related sounds would differentiate from the *Natural* sounds due to the mental representation of motor characteristics, that was indeed more pronounced. We further aimed to investigate whether statistical differences also apply to the comparison between *Musical* and *Artificial* as it has been previously reported; based on the assumption that the sounds of daily-used objects might trigger more the response of action compare to the musical instruments (in non-musicians). Our results did not yield any significant differences in source space analysis, however, in the connectivity analysis, even though the *Musical-versus-Natural* and *Artificial-versus-Natural* demonstrated similar connectivity patterns, the strength of the interconnections (as given by the thickness of the edge) was different. For instance, in the *Artificial-versus-Natural* network, the inter-hemispheric prefrontal edge and the STG-PCC edge demonstrated stronger interconnections among the rest, whereas the *Musical-versus-Natural network* yielded a more distributed intensity across the interconnections with smaller values. A previous study (De Lucia et al., 2009) has shown differential discrimination between musical and tool sounds only after 300 ms stimulus onset. On this basis, it might be that the discrimination of broader categories of sounds, such as between living and man-made sounds already occur on early responses, though discrimination of subcategories could be followed by later responses. It would be interesting in the future to investigate later time intervals in which higher-level conceptual processes might be needed for the discrimination of man-made subcategories. It should be mentioned though that the *Artificial* category in our study, contained sounds that are generally man-made objects, however, some of them were less manipulative (e.g., ambulance siren). The absence of a stricter categorization based on a behavioral assessment that would divide the objects based on familiarity and the frequency of use, might have limited our interpretation regarding the significant differences between sound categories. The examination of the cortical regions in response to sound familiarity, recognizability and attention might give insights on the role that the “what” auditory neuronal pathway has in sound processing. Previous studies have shown the importance of sound novelty, task demands and attention in sound discrimination (Levy et al., 2001, 2003). However, others argue that the discrimination of living and man-made objects is present independently of behavioral proficiency, and hence familiarity (De Lucia et al., 2012), when correctly categorized sounds were compared with incorrectly categorized sounds as well as independently of consciousness (Cossy et al., 2014) when comatose patients were examined. This is still on debate in the literature and should be addressed in future studies.

A possible limitation of the current study might be that the categorization of complex sounds could be confounded by the physical differences of stimuli (Belin et al., 2000; Staeren et al., 2009), something that by purpose was not controlled here, in order to avoid any distortion in the quality of the sounds. However, natural sounds differ from human-made and more synthetic sounds by nature which we cannot manipulate. According to Theunissen and Elie’s (2014) review, the natural sounds consist of statistical properties that follow a power law relationship and are optimally encoded in the ascending auditory processing system in contrast to the sounds with more random and flat envelope power spectra (such as the sounds in the Artificial category). Nevertheless, similar categorization of sounds has been administered in the past and our results are in consistence (Murray et al., 2006).

The results of our study suggest that the dissociation between living and man-made objects, is based on distinct neuronal processing. However, the reason of this phenomenon is still questioned. From another perspective, it might be that sounds have been conceptually specialized in the processing of different categories of sounds due to evolutionary adaptation; a theory that has been strongly argued for the visual system, as well (Caramazza and Mahon, 2003). According to that, distinct cortical pathways corresponding to different categories of sounds, have been evolved analogous to the environmental sounds, which humans have experienced over the years. This would be in agreement with the previous mentioned review that suggests optimally neural encoding over the natural sounds compare to the sounds of human-made machines (Theunissen and Elie, 2014). From this perspective, listening to the sounds of nature would elicit weaker activation of brain in comparison to the more “modern” tool sounds (e.g., phone ringing), since we are evolutionary more adapted to the natural sounds and this in turn would require less cognitive effort (Buckner et al., 2008). This would in turn give some explanation on the abovementioned findings, where the man-made sounds showed stronger brain activation compare to the “living” objects. Furthermore, the functional connectivity maps showed that within the man-made category, there might be “key” connections for *Artificial-versus-Natural* relative to *Musical-versus-Natural*, even though the nodes remained the same for both comparisons. This could also explain cases of semantic impairments, where patients are impaired in a very specific category, while the remaining categories within the same domain are spared (McCarthy and Warrington, 2016; Muhammed et al., 2018). Therefore, it seems very probable that the perception of auditory objects relies on a large-scale distributed system, which follows distinct neuronal pathways, dissociated on the basis of the weight that each node has in the network. Based on our findings, this assumption cannot be clearly answered, though it gives rise for upcoming examination. A connectivity analysis involving also the common activated regions might provide a better picture to this assumption, however, due to the fact that this requires a different analysis, it is recommended for future study.

In general, the fact that the processing of auditory stimuli engages regions beyond the auditory cortex, such as anterior temporal and frontal lobe (Maeder et al., 2001; Alain et al., 2008), is well documented. Similar to our cortical network analysis results, recent neuroimaging studies suggest that a network of fronto-temporo-parietal regions contributes to semantic processing (for review see Thompson-Schill, 2003; Patterson et al., 2002). This network has been proposed to be associated with the perception of both auditory and visual object identification (Goll et al., 2010; Hunter et al., 2010; Brefczynski-Lewis and Lewis, 2017). However, what is not well documented yet is how these brain responses are functionally connected. Our connectivity study underpins the connectivity of brain regions within sound discrimination. In order to obtain a better understanding of brain auditory categorization, it is not sufficient to investigate only the activated regions in isolation, but rather to understand how these regions interact. In this aspect we believe that our results are valuable for better understanding of the human brain in sound discrimination.

## Conclusion

The present study demonstrated an enhanced brain network of man-made related sounds (*Musical* and *Artificial*) when compared to *Natural* sounds. So far the literature has provided simple brain activation maps. We additionally showed how these differentially activated brain regions are functionally connected and linked to the respective cognitive processes. We replicated previous findings supporting the engagement of other modalities beyond the auditory, to be involved in the processing of sound stimuli, as soon as this reaches the level of object representation. This in turn seems to be based on semantic categorization of the stimulus, following distinct neuronal pathways for living versus man-made objects. In addition to previous studies that investigated only the cortical activation to different sound categories, we demonstrated significant differences in the functional connectivity between the cortical sources involved in the processing of the different sound categories.

## Data Availability Statement

The datasets generated for this study are available on request to the corresponding author.

## Ethics Statement

The studies involving human participants were reviewed and approved by the Medical faculty of the University of Münster. The patients/participants provided their written informed consent to participate in this study.

## Author Contributions

VS contributed to the data recruitment, data analysis, and wrote the first draft of the manuscript. EP and NC involved in the statistical analysis. KM and AW involved in the informatic support. CD, DK, and CP contributed to the manuscript revision. All the authors read and approved the submitted version of the manuscript.

## Conflict of Interest

The authors declare that the research was conducted in the absence of any commercial or financial relationships that could be construed as a potential conflict of interest.

## Footnotes

1 www.freesounds.org

2 www.soundbible.com

3 www.zapsplat.com

4 www.audacityteam.org

5 www.nch.com.au

6 www.neurobs.com

7 www.fil.ion.ucl.ac.uk
